# Impact of Mixing on Content Uniformity of Thin Polymer Films Containing Drug Micro-Doses

**DOI:** 10.3390/pharmaceutics13060812

**Published:** 2021-05-29

**Authors:** Guluzar G. Buyukgoz, Jeremiah N. Castro, Andrew E. Atalla, John G. Pentangelo, Siddharth Tripathi, Rajesh N. Davé

**Affiliations:** Otto H. York Department of Chemical and Materials Engineering, New Jersey Institute of Technology, Newark, NJ 07102, USA; gg275@njit.edu (G.G.B.); jnc24@njit.edu (J.N.C.); aea33@njit.edu (A.E.A.); jgp26@njit.edu (J.G.P.); st699@njit.edu (S.T.)

**Keywords:** polymer thin films, low drug concentration, content uniformity, homogeneity, critical process parameters (CPPs), Taguchi design, mixing effectiveness

## Abstract

The impact of mixer type and critical process parameters (CPPs) on critical quality attributes (CQAs), including the drug content uniformity (CU) of slurry-cast polymer films loaded with micro-sized poorly water-soluble drugs were investigated. Previously untested hypothesis was that the best mixer at suitable CPPs promotes uniform drug dispersion within film precursors leading to acceptable dried-film CU at low, ~0.6 wt% drug concentrations. Taguchi design was utilized to select the best of three mixers; low-shear impeller, high-shear planetary, and high-intensity vibrational, for dried-film drug concentration of ~23 wt%. As-received fenofibrate, a model poorly water-soluble drug (~6 µm) was directly mixed with the hydroxypropyl methylcellulose (HPMC) and glycerin aqueous solution. Impeller and planetary mixers yielded desirable film relative standard deviation (RSD), while vibrational mixer could not. For the lowest dried-film drug concentration of ~0.6 wt%, only planetary mixer yielded RSD <6%. The precursor drug homogeneity was a sufficient but not a necessary condition for achieving dried-film RSD <6%. Thus, proper selection of mixer and its CPPs assured desirable film CQAs. However, minor drug particle aggregation was identified via re-dispersion testing which also led to incomplete drug release.

## 1. Introduction

Recent research has established polymer-based films as a drug delivery platform with several advantages over traditional solid oral dosage forms [1,2,3,4,5,6,7,8,9,10]. The polymer films are considered to be patient compliant and self-administrable, particularly among pediatric and geriatric patients. Moreover, they are recognized as an inherently robust platform since the film-forming polymer acts as a flexible carrier for the drug particles. More importantly, it was shown that the polymer films offer a promising delivery platform for poorly soluble drugs, leading to numerous papers on the incorporation of poorly water-soluble drugs into polymer films [4,7,8,10,11,12,13,14,15].

Amongst the preparation techniques of the films [14,16,17], slurry casting [1,2,3,4,5,8,15,17,18,19,20,21] has attracted significant attention for delivery of poorly water-soluble drugs. In this approach, crystalline drug particles are incorporated into a polymer solution by mixing, followed by casting, and drying. Since the process is solventless, the physical stability of drug particles is naturally preserved throughout the process [3]. In addition, unlike typically thicker films resulting from melt extrusion technique [16,22], the thin films less than 100 µm could be produced via slurry-casting technique. Thinner films require the use of small-sized drug particles for achieving more uniform drug particle distribution within the film matrix. Use of the fine particles poses challenges in manufacturing for achieving uniform drug distribution due to fine particle cohesion. However, if that challenge can be overcome, it could also help with faster drug dissolution rates owing to the larger exposed surface area from fine particles [23,24,25]. Thus, a major concern for the slurry casting technique has been the aggregation of primary drug particles, negating potential advantage of product uniformity. Therefore, recent efforts to both circumvent these problems and improve critical quality attributes (CQAs) of films have focused on particle engineering methodology. Beck et al. [26] produced low-micron size drug particles using liquid-antisolvent precipitation. With the help of stabilizers, they controlled the particle agglomeration and achieved fast dissolving polymer films for griseofulvin, a poorly water-soluble drug. Later, Krull et al. [5] produced wet-milled drug nanoparticles for several poorly soluble drugs and incorporated them into polymer-based films. All the film samples loaded with drug nanoparticles exhibited excellent content uniformity with relative standard deviation (RSD) of <4% in terms wt% of the drug and demonstrated fast dissolution even for very poorly water- soluble drugs [5].

In the series of research articles [6,17,18,20], a novel approach based on the direct incorporation of dry surface-modified micro-sized particles into polymer films for delivery of poorly water-soluble drugs was established [6]. The results revealed that the surface-modified particles promoted uniform dispersion of drug particles within the film matrix and led to better performance in drug particle re-dispersion, complete drug release, and drug content uniformity [6]. Thus, the overall conclusion of these papers on slurry casting is that the combination of particle engineering that produces stabilized nano or low-micron sized drug particles in wet or dry form, provide better drug distribution leading to desirable CQAs, if drug particles are fine (<10 µm) in combination with either higher viscosity film precursors or high shear mixing devices. Unfortunately, to date the role played by mixing in preparing low drug loaded film precursors at low drug concentrations, i.e., equivalent to dried-film drug concentration of 3 wt% or lower, has not been investigated. In fact, that is the most challenging issue in achieving excellent drug content uniformity and particle re-dispersion at low drug concentrations. In addition, the assessment of the homogeneity of the film precursor and how that impacts the drug CU in the dried films has also been ignored. Therefore, the present work aims to fill this gap by investigating the role played by the mixers in both the drug content uniformity in dried-films and the homogeneity of the film precursors.

Previous studies have shown that when the industrially relevant impeller mixer was used along with the use of surface stabilization via polymer and surfactant addition for nano-suspensions, the use of higher viscosity polymer solutions was necessary, due to the higher induced shear [19,27]. As a major advance, a comprehensive study using dry micronized drug powders at ~20 wt% drug concentration of fenofibrate demonstrated that powder surface modification via dry coating with hydrophilic silica M5P led to very good films even without the need for both the higher viscosity film precursors or higher intensity planetary mixer. Unfortunately, that work did not examine low drug concentration and also demonstrated that when surface modification via dry coating was not done, higher viscosity film precursors and/or higher intensity planetary mixer were necessary. To properly identify the role of mixing, the current work did not consider surface modification or stabilization and further, did not employ high film precursor viscosity levels as will be discussed later.

Overall, the main hypothesis of this work is that by properly selecting mixing conditions through the preliminary design of experiments (DoE), those conditions may also be used for producing films at very low drug loadings, i.e., 3 wt% and 0.6 wt%, while achieving desirable polymer film product CQAs including drug content uniformity levels, using as-received micro-sized poorly water-soluble active pharmaceutical ingredient (API) particles. An important question remains if the as-received API size is suitable for achieving the desired drug content uniformity at the lowest drug loading of 0.6 wt%. Towards that objective, the guidance on the smallest particle size for a given standard deviation that could assure the selected low dosage assuming no particle aggregation was used as per the nomograph from [28]. Accordingly, for the intended drug particle size to be used (~6 µm), desired CU is possible for the lowest drug loading of 0.6 wt%. Given that assurance, the plan of the study is discussed next. In what follows, in addition to a conventional low-shear impeller, a high shear planetary mixer and a high-intensity vibrational mixer were used. As-received fenofibrate, ~6 µm, was selected as a model poorly water soluble Biopharmaceutics Classification System (BCS) class II drug, selected for consistency with the previous work. Low molecular weight hydroxypropyl methylcellulose (HPMC) was used as the film former as well as a stabilizer, while glycerin was used as a film plasticizer.

In the first part of the investigation, for three selected mixers, their critical processing parameters (CPPs), i.e., mixing intensity and mixing time, were investigated through Taguchi L9 orthogonal array DoE. The purpose was to identify the top-performing mixers and their CPPs while utilizing a higher level of drug concentration, i.e., 23 wt%, comparable to previous work. Before forming dried films, an initial assessment for the uniformity of drug distribution was done via measuring drug homogeneity within the resulting film precursors. The dried films were characterized in detail by assessing their mechanical properties, drug particle aggregation through optical microscopy, drug particle re-dispersibility that is a measure of the extent of irreversible agglomeration, drug dissolution behavior, and most importantly, the drug content uniformity. Two top-performing mixers and their respective operating conditions were used for the next stage of the investigation. Using those mixers at their best processing conditions, film precursors were mixed followed by producing films at two lower drug loadings of ~3 wt% and ~0.6 wt%. The resulting dried films were evaluated for their drug content uniformity to help test the proposed hypothesis.

## 2. Materials and Methods

### 2.1. Materials

As-received, crystalline, micron-sized (primary particle size of ~6 µm) fenofibrate (AR-FNB; Jai Radhe Sales, Ahmedabad, India) was selected as model Biopharmaceutics Classification System (BCS) class II drug [29,30,31,32]. Low molecular weight hydroxypropyl methylcellulose (HPMC; Methocel E15 Premium LV, Mw ~40,000; The Dow Chemical Company, Midland, MI, USA) was used as a stabilizer and film-forming polymer. Glycerin (Sigma–Aldrich, St. Louis, MO, USA) was used as a film plasticizer. An aqueous solution of sodium dodecyl sulfate (SDS) (Sigma-Aldrich, St. Louis, MO, USA) received in powder form was prepared at 7.2 g/L concentration. This solution was used for assay testing. In addition, the dissolved SDS was also used as the surfactant in the dissolution media for all the dissolution tests. All other materials were used as received.

### 2.2. Preparation Methods

#### 2.2.1. Preparation of Film Precursor Suspension

Formulations for the polymer solutions and precursor suspensions are listed in Table 1. The polymer solution was prepared as per the Dow protocol for HPMC E15LV polymer, which involves heating deionized (DI) water to 30 °C before adding glycerin (4 wt%) while stirring magnetically. Heating was continued and upon reaching 90 °C, the required amount of HPMC (12 wt%) was slowly added under continued stirring. Once the polymer was fully dispersed, the solution was cooled down to room temperature which was then used to prepare the placebo films. Although the minimum precursor viscosity for a reliable film formation has been suggested to be ~5000 cP [2,19]. For consistency with the previous work [6,19], 12 wt% HPMC polymer solution was used. It met the minimum critical viscosity, achieving a level of ~12,000 cP. Even this level is relatively low as compared to the high viscosity level recommended for improved film CQAs [6]. However, for better discernment of the impact of mixing parameters on film CQAs, the use of a much higher viscosity precursor was avoided.

Film precursor to prepare the slurry films involved mixing of the dry micro-sized FNB with the polymer solution at various compositions listed in Table 1, including the target drug concentrations (wt%) in the final dried-film product. The mixing was performed using three different mixers, standard impeller mixer (RW16, IKA, Wilmington, NC, USA) providing low-shear, planetary centrifugal mixer (Thinky, Laguna Hills, CA, USA) providing high-shear forces, and high-intensity vibrational (V) mixer (Laboratory Resonant Acoustic Mixer, LabRAM; Resodyn Acoustic Mixers, Inc., Butte, MT, USA), providing intense yet low-shear mixing. The range of critical processing parameters (CPPs) i.e., mixing intensity and mixing time, affecting the mixing performance were selected as per the specifications for each mixer type. These ranges were used for Taguchi design L9 orthogonal array design of experiments, performed using MINITAB statistical software version 19 (Minitab Inc., State College, PA, USA). A Taguchi design, detailed in Section 2.4, was used because it allows delineating the impact of each mixing parameter on the final product through minimal trials, hence saving time and resources [33]. The experimental design is presented in Table 2 with additional details in the Supplementary Materials, Table S1.

#### 2.2.2. Preparation of Polymer Films Containing Drug Particles

The precursor suspensions prepared as per conditions for three different mixers in Table 2 were cast onto a plastic substrate (Scotchpak TM 9744, 3M, St. Paul, MN, USA) with a doctor blade (Elcometer, Rochester Hills, MI, USA) at a constant casting thickness of 1 mm using a Lab-Cast Model TC-LC Tape Caster (HED International, Ringoes, NJ, USA). The wet films were dried at 50 °C in the Tape Caster, which provides simultaneous conductive and convective heating. Once the film was dried, it was peeled from the substrate and stored in a sealed plastic bag.

### 2.3. Characterization Methods

#### 2.3.1. Particle Sizes

The particle size distributions (PSDs) of dry AR-FNB particles were measured using a Sympatec Helos/Rodos pressure dispersion and laser diffraction system (Sympatec, Pennington, NJ, USA). The d_10_, d_50_, and d_90_ values of the particle size statistics were reported at the dispersion pressure of 1.0 bar based on Fraunhofer and Mie scattering theories [34]. To assess the impact of mixing on particle dispersion, the redispersion of FNB from dried-films was carried out using a previously established protocol [4,5]. Accordingly, 2 to 3 circular film punches, about 0.7 cm^2^ in area, were dispersed into 3 mL of deionized water, followed by vortex mixing (Fisher Scientific, Waltham, MA, USA) for 3 to 5 min at 1500 rpm. The FNB particle sizes in the dispersed suspension were measured in wet mode via a Coulter LS 13320 Laser Diffraction Particle Size Analyzer (Beckman Coulter, Miami, FL, USA), which employs a polarized intensity differential scattering (PIDS) obscuration water optical model [4].

#### 2.3.2. Drug Content and Uniformity

##### Homogeneity Testing for Precursor Suspension

**Figure 1:**
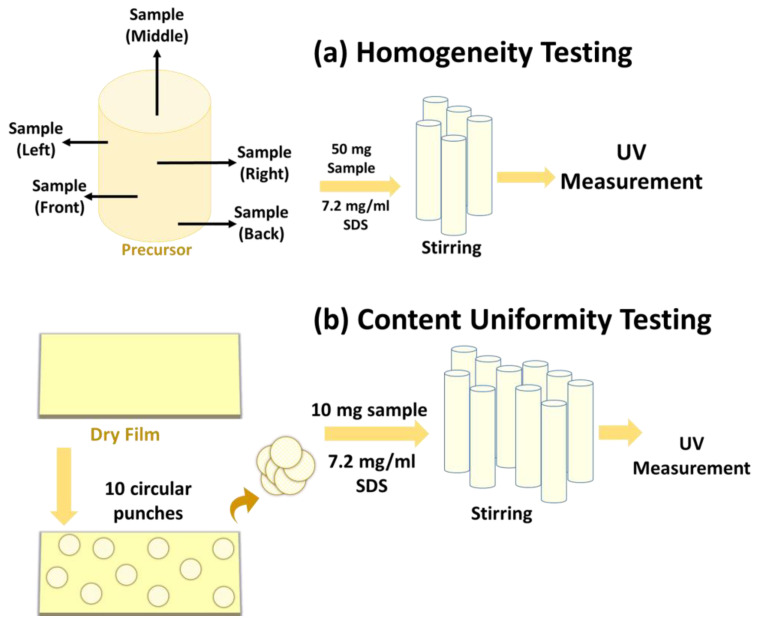
Schematic of the procedures: (**a**) Homogeneity testing from precursor suspension; (**b**) Drug content uniformity (CU) testing from the dried-film.

To assess the homogeneity of drug dispersion in precursors, samples were removed from five different locations of the precursor container, namely middle, left-upward, right-upward, front bottom, and back bottom. Figure 1a presents the schematic of the sampling and procedure for the homogeneity test. To maintain consistency between the film precursor sample amount and the content uniformity testing for the dried-films, their theoretical drug amounts were kept the same. Accordingly, the precursor sample was 50 mg, which was equivalent to the theoretical drug amount in a test sample sized ~0.7 cm^2^ of the dried-film. The collected samples from precursor suspension were transferred to solvent bottles containing 50 mL of 7.2 g/L SDS solution and stirred for at least 3h to allow complete dissolution of the drug particles. Subsequently, the drug amount was tested using a Thermo Scientific Evolution 300 UV–Vis spectrophotometer (Thermo Fisher Scientific Inc., Waltham, MA, USA) at a wavelength of 290 nm. The relative standard deviations (RSDs%) for the corresponding drug amounts were calculated, with lower RSD% values being indicative of a homogeneous product.

##### Content and Uniformity Testing for Dried-Films

The drug content and uniformity of the dried films were determined as per previous protocol [5], depicted in Figure 1b. Ten circular film samples ~0.7 cm^2^ in area were removed from random locations on the films. 100 mL and 20 mL of 7.2 g/L SDS solution were used to dissolve the film samples at high and low drug concentrations, respectively, and stirred continuously for a minimum of 3 h. To measure the drug amounts in the samples, a similar procedure as in the homogeneity test was used including the measurement of the UV absorbance. The results were expressed as relative standard deviation (RSD%) and acceptance values (AV) following the established protocols [35]. The thickness of the samples was measured using a digital micrometer (Mitutoyo Corporation, Kawasaki, Kanagawa, Japan). The RSD% values of weight percentage of the drug in the film and drug dose per unit area were also calculated.

#### 2.3.3. Mechanical Properties

The mechanical property analysis of the dried-films was conducted using a TA-XT Plus Texture Analyzer (Stable Microsystems, Godalming, UK). Four to six rectangular samples having dimensions of 50 mm × 15 mm were cut from the dried film. The average thickness along with the standard deviations was reported. The film samples between the clamps were stretched at a constant rate (1 mm/s) until they fractured, to obtain the stress-strain curve, from which film tensile strength (TS) and percent elongation at break (EB%) were calculated.

#### 2.3.4. Light Microscopy

To examine the morphology of the FNB particles after mixing and drying, the samples from precursor suspensions and dried-films were observed under a polarized light microscope (Carl Zeiss Microscopy, LLC, Oberkochen, Germany). While rectangular pieces of dried-films were directly used for imaging, for film precursor imaging, one drop was deposited onto a glass slide and observed at 10× magnification.

#### 2.3.5. Dissolution

The dissolution test was performed using USP IV, a closed-loop flow-through cell dissolution apparatus (Sotax, Aesch, Switzerland) following previously reported procedure under sink conditions [5,36]. Six circular film samples of ~0.7 cm^2^ in area were positioned inside the cells within 5 g of 1 mm glass beads. The dissolution medium (7.2 g/L SDS solution, 200 mL) [6] was circulated through the cells at a flow rate of 16 mL/min. The temperature was maintained at 37 ± 0.5 °C throughout the test. Percentage FNB released was determined based on the drug content assay as discussed in Section 2.3.2. The actual amount of FNB in terms of drug concentrations in each sample was calculated for each film formulation, taking into account the weight of each sample. The dissolution results are reported as dissolved FNB (%) as a function of time.

#### 2.3.6. Thermo-Gravimetric Analysis (TGA)

Thermo-gravimetric analysis (TGA) of AR-FNB particles and the dried-films was carried out using TGA/DSC1/SF STARe system (Mettler Toledo Inc., Columbus, OH, USA). Samples in the amount 5 to 8 mg were placed in a ceramic crucible and heated in a nitrogen atmosphere from 25 °C to 250 °C at a constant rate of 10 °C/min.

#### 2.3.7. X-ray Diffraction (XRD)

The X-ray diffraction (PANanalytical, Westborough, MA, USA) was used to determine the solid-state of the drug particles before and after processing in the films. AR-FNB particles, placebo film, and the films at 23 wt% drug concentration were scanned at a 2θ angle in a range of 5–35° at a rate of 0.01 s^−1^.

#### 2.3.8. Viscosity

The apparent shear viscosity of the precursor suspension at 5 wt% FNB concentration, corresponding to dried-film FNB concentration at 23 wt% was tested with an R/S-CC plus rheometer (Brookfield Engineering, Middleborough, MA, USA), combined with a shear rate controlled coaxial cylinder (CC25) and Lauda Eco water jacket assembly (Lauda-Brinkmann LP, Delran, NJ, USA) for temperature control. The viscosity values were recorded at a low shear rate of 2.2 s^−1^ at 25 ± 0.5 °C.

### 2.4. Statistical Analysis via the Taguchi Method

Taguchi design was used to determine the proper experimental settings with the given design parameters by creating an orthogonal array, which greatly reduces the number of experimental configurations [37]. The method utilized signal-to-noise (S/N) ratio as the important quality characteristic of selection, where the noise is defined as the factor causing deviations from the target value of the functional characteristic [38,39]. For defining the S/N ratio, the smaller the better approach [39] was used since the product uniformity was evaluated via RSD% values, which are desired to be as small as possible. A standard table, known as a Taguchi L9 orthogonal array with three levels and three factors, (shown in Table 2) was used for the design of the experiment employing Minitab software version 19. Three film critical processing parameters, CPPs, mixer type, mixing intensity, and mixing time, at three levels of their mixing conditions; high, medium, and low, were employed for the design of experiment (see Supplementary Materials, Table S1) [6,40].

## 3. Results

### 3.1. Drug Crystallinity

**Figure 2:**
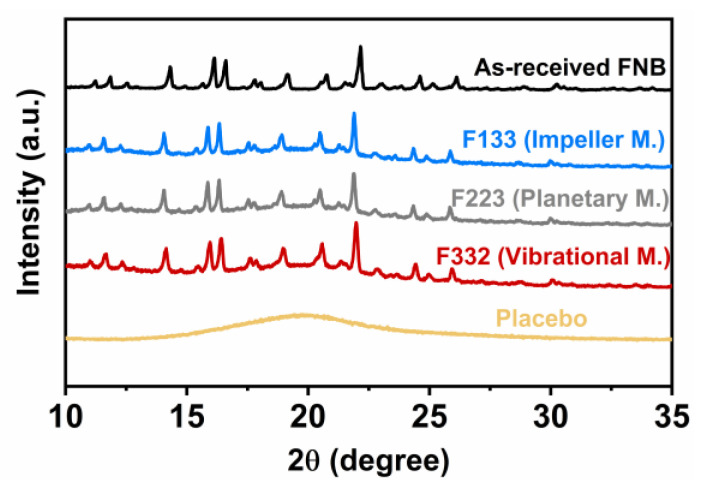
XRD patterns of AR-FNB powder, placebo film, and the films containing ~23 wt% FNB concentration processed with the mixing conditions of F133, F223, F332.

The XRD patterns of AR-FNB powder, placebo film, and the films loaded with AR-FNB particles are shown in Figure 2. AR-FNB particles exhibited sharp diffraction peaks, referring to the crystalline form of FNB. The characteristic peaks of FNB appeared in the diffraction pattern of all the film samples except the placebo film. That suggested the crystalline state of FNB particles was maintained throughout the film preparation including the mixing at varying conditions, film casting, and drying.

### 3.2. Thermo-Gravimetric Analysis (TGA)

**Figure 3:**
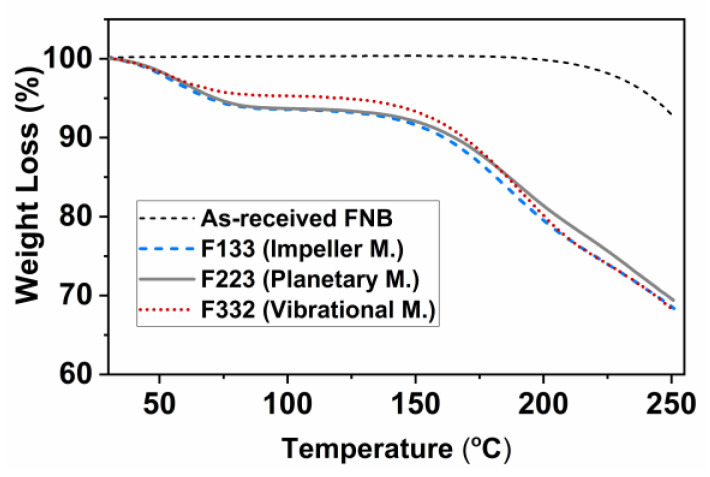
TGA curves of AR-FNB particles and the films containing ~23 wt% FNB concentration processed with the mixing conditions of F133, F223, F332.

The results of TGA analysis are shown in Figure 3. No weight loss was observed up to 150 °C for dry AR-FNB particles, implying no degradation issue. The films made with hydrophilic polymer solution using different mixers; impeller, planetary, and high-intensity vibrational (V), exhibited weight loss of about 5 to 6 wt% at 100 °C. That indicated free or bound water [41] within the polymer film structure owing to the hydrophilic nature of polymer used or entrapped water molecules during film preparation. A weight loss of about 7 to 9 wt% was observed when heating the samples to 150 °C. That could be attributed to the loss of glycerin, which is in agreement with previous observations [19]. In conclusion, TGA results indicated that the drying for the film product was as desired since the moisture content in dried-films was found to be about 5 to 6 wt%, eliminating any bias over moisture-induced variations during film characterization.

### 3.3. Drug Particle Size and Recovery after Redispersion

**Figure 4:**
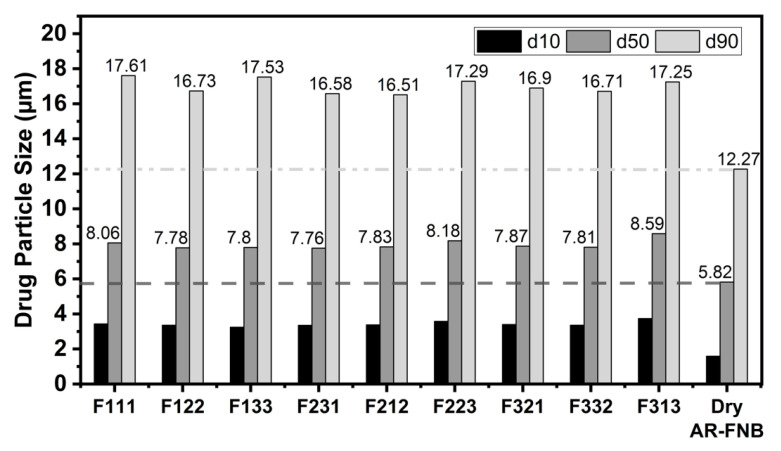
Particle size distribution of AR-FNB after re-dispersion from dried-films containing ~23 wt% drug concentration along with the particle size of dry AR-FNB.

The measured primary particle size of dry AR-FNB particles was d_50_: 5.82 µm and d_90_: 12.27 µm. The particle sizes for as-received and redispersed from dried-film processed at varying mix conditions are shown in Figure 4. The redispersed particle sizes from all the dried-films ranged between 7.78–8.59 µm for d_50_ and 16.51–17.61 µm for d_90_. Generally, decreasing the mixing intensity or mixing time, increased the re-dispersed particle size as compared with the primary particle size (d_90_) of dry AR-FNB. Nevertheless, regardless of the mixing conditions, the d_90_ values of the re-dispersed particles from each film (see Figure 4) increased by about 35–44%, which suggested some degree of drug aggregation. This is most likely due to the micro-sized drug particles used, which often possess high relative cohesion [34,42] and are prone to aggregate. The resulting aggregation issue has been previously reported by Zhang et al. [6] who used untreated micro-sized drug particles in film preparation.

### 3.4. Digital Microscopy

**Figure 5:**
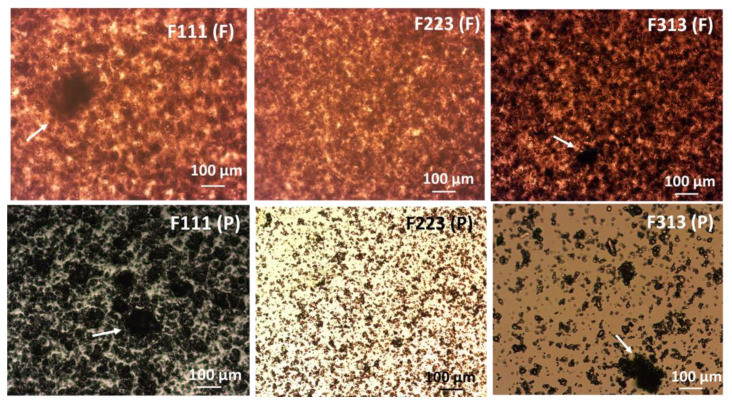
Optical microscopy images of dried-films (F) and precursor suspensions (P) processed at different mixing conditions, the first row from left to right; F111 (F), F223 (F), F332 (F), and second row from left to right; F111 (P), F223 (P), F332 (P).

Optical microscopy images captured from the precursor suspensions and dried films are shown in Figure 5. The selected process parameters for each mixer type are discussed in the following section. The drug particle sizes observed in the images, which are larger than the primary particle size of AR-FNB were considered as drug aggregates. Thus, some extent of drug aggregation was present in both precursor suspensions and dried films. However, the microscopy images of the film precursors mostly exhibited larger aggregates compared to that of the dried films. That is most likely due to easier visualization from aqueous precursor suspensions, and particle movement and aggregation during sample preparation. The films processed at low mixing intensity, F313 and F111, showed large and irregularly distributed aggregates/lumps sized about 141 and 182 µm, respectively, whereas the film processed with a high mixing intensity, F223 showed a much smaller drug aggregate i.e., ~52 µm. This implies that the higher mixing intensity could be more effective in dispersing the particles as compared to the longer mixing time, since F313 and F223 are both high-intensity mixers. Surprisingly, in the sense of dispersing the particles, F313, processed with the high-intensity vibrational mixer showed similar performance as F111 being processed with the low-shear mixer, suggesting that its mode of operation does not facilitate particle dispersion as good as the planetary mixer. In summary, the mixing conditions including mixer type and mixing parameters have a significant impact on particle dispersion, and careful study was required for adjusting these conditions to achieve desired uniformity.

### 3.5. Drug Content Uniformity

#### 3.5.1. High Drug Concentration (23 wt%)

**Figure 6:**
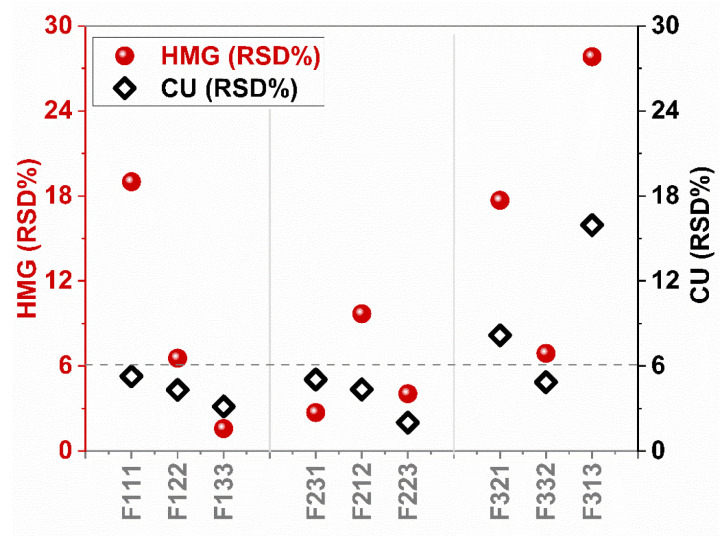
Comparison of homogeneity and content uniformity tests with respect to RSD% of the corresponding drug amounts. Final drug concentration: ~23 wt%. The horizontal line indicates the acceptable RSD for HMG and CU.

The mixing effectiveness on drug content and uniformity was measured through RSD% of the drug amount per sample from precursor suspensions, as homogeneity (HMG), and as content uniformity (CU) for dried-films, at 23 wt% drug concentration (see Section 2.3.2 for the procedure details). The results are presented in Figure 6, and Table S2, Supplementary Materials. For each mixer type, as the mixing intensity increased, the homogeneity of the precursor improved, regardless of the mixing time i.e., RSD% of F231 is 2.7 and of F223 is 4.04. Although this observation was valid for the high-intensity vibrational (V) mixer, unfortunately, all the resulting precursors at varying mixing conditions, F313, F321, and F332, showed RSD% greater than 6%, which is above an acceptable uniformity. Moreover, the low-shear impeller mixer performed better than the vibrational mixer in the sense of resulting homogeneity of the precursors (see Figure 6). The planetary mixer outperformed the other mixers, and the only precursor processed at the lowest mixing intensity (F212) was out of the acceptable uniformity range. In addition, these results conveyed an important message that the high energy input through mixing conditions may not always produce homogeneous precursors. This inference was further evaluated by comparing the homogeneity of the precursors with drug content uniformity of the corresponding dried films (see Figure 6). The most noteworthy outcome is that RSD% values reduced when the products were cast and dried to form films. For example, the precursor of F111 showing RSD% of ~19 resulted in a contently uniform final product with RSD below 6%. It is important to recognize that the tested film samples are rather small, i.e., ~0.7 cm^2^ in area, as compared to typical dosage size for films hence such outcome for the dried-film RSD is excellent. These results confirm that the film format is capable of producing uniform products owing to the involvement of additional process steps of casting and drying even if the homogeneity is a little less than adequate [5]. Another discernable trend is that when the homogeneity of the precursor is in the acceptable range, i.e., RSD <6%, that seems to assure the uniformity of the final products (see Table S2, Supplementary Materials), regardless of the mixer type used. That implies that a homogeneous precursor could be the sufficient conditions for the content uniformity of final products (films), opening a pathway to utilize PAT tools at the mixing stage in the future.

Variations in film properties including thickness, drug concentration (wt%), drug dose (mg/cm^2^), as well as acceptance values (AVs) are evaluated and presented in Table 3. Overall, F313 and F321, both for vibrational mixer, showed unacceptable uniformities in almost all the film properties listed above, while at all the mixing conditions, planetary mixer (F231, F223, F212) could produce uniform films. As mentioned before, F111 produced non-homogeneous precursor but contently uniform dried film. However, the product showed a high AV of 25.26. This could be explained by some loss of the drug particles due to their sticking to the walls of the mixing vessel during the mixing process, leading to a lower drug concentration than the label claim (see Table 3). Thus, these mixing parameters were inadequate for properly mixing the drug and polymer solution. However, when the mixing time and intensity were increased, i.e., F122 and F133, uniform films were achieved. The analysis of these results revealed that longer mixing may not be required when mixing intensity was sufficient, as evident from F122 and F332 (see Table 3). This is confirmed by the main effects plot from Taguchi design, where mixing intensity was found to be more important than the mixing time, further discussed in the next paragraph. For the sake of brevity, the results of response table for S/N ratios are provided in Table S3, Supplementary Materials.

Overall outcomes of the mixer performance at different intensity and times, suggested that while a high-shear planetary mixer is capable of producing uniform products at all mixing conditions, the high-intensity vibrational mixer required more intense mixing conditions. While both are high-intensity mixers, they have differing mechanisms and may respond differently over the typical range of the intensities employed. For instance, the planetary mixer simultaneously applies revolution and rotation with the rotation axis at 45°, which effectively agitates the mixture without the agitator blades [43]. Further, the rotational speed on particle dispersion has been acknowledged [44]. In contrast, the high-intensity vibrational mixer employed vertical motion [45]. At lower intensities, the mixture was more likely to undergo rigid body motion instead of intense convective mixing hence the mixing intensity should not be lower than 60 G for precursor suspension with ~12,000 cP viscosity. Likewise, the low-shear impeller mixer required both higher rotational speed and mixing times. Based on these outcomes from Taguchi design of experiments, a subsequent investigation will focus on utilizing the impeller and planetary mixers, picking the mixing conditions giving the most uniform films, F133 and F223.

#### 3.5.2. Low Drug Concentrations (3 wt% and 0.6 wt%)

Next, assessment of mixing effectiveness was done for films at low drug concentrations of 3 wt% and 0.6 wt% using as-received low micro-sized drug particles. The results presented in Table 4 show that for the impeller mixer, decreasing the drug concentration decreased the product uniformity, since RSD% and AV of the films increased. Particularly, RSD% and AV at the lowest drug concentration were not within acceptable limits. However, as mentioned earlier, since the tested film samples were rather small, i.e., ~0.7 cm^2^ in area, as compared to typical dosage size for films, these outcomes for the dried-film RSD are reasonable. Most interestingly, even for testing on small sample sizes, the planetary mixer exhibited an excellent performance. It consistently produced uniform products even for the films containing micro-doses as low as ~70 µg/cm^2^.As discussed before, this level of drug content uniformity is theoretically possible with the given particle size assuming no agglomeration at this low drug concentration conditions as per the nomograph in Huang et al. [28]. Hence these results demonstrate that the mixing afforded by the planetary mixer led to largely deagglomerated particles and provided adequacy to assure the product uniformity even at these very low drug concentrations. These results also indicate that the film format along with adequate mixing offers a robust manufacturing platform technology that could be used for high quality manufacturing of micro-dosed polymer films.

### 3.6. Mechanical Properties

The mechanical properties of the films including tensile strength and elongation at break (EB%) are shown in Table 5. Although there are no established standards for the mechanical properties of films, they must have a certain mechanical resilience for handling and packaging [2]. The films processed at different mixing conditions (see Table 2) exhibited similar TS values, but small differences in EB%. Generally, the films having more uniform drug dispersion, which was achieved by increasing the mixing intensity or time, had higher EB% values. For example, EB% enhanced from 10.0 to 15.4 for improved mixing conditions, F332, from F321. Similarly, enhancements are observed between F223 from F212 and F122 from F111. Such results are in agreement with previous work that indicated that disruptions in the film matrix stemming from non- uniformities such as particle agglomeration may create stress spots and cause easy rupturing of the films [46]. These results were further evident in the digital images of the films, see Figure S1, Supplementary Materials, where drug lumps or aggregates appeared on the film surface. Repka et al. [46] also observed similar phenomena in polymer films while working with hydroxypropyl cellulose (HPC) polymer, where increases in drug agglomeration were linked to the decrease in film elasticity and flexibility.

### 3.7. Dissolution

**Figure 7:**
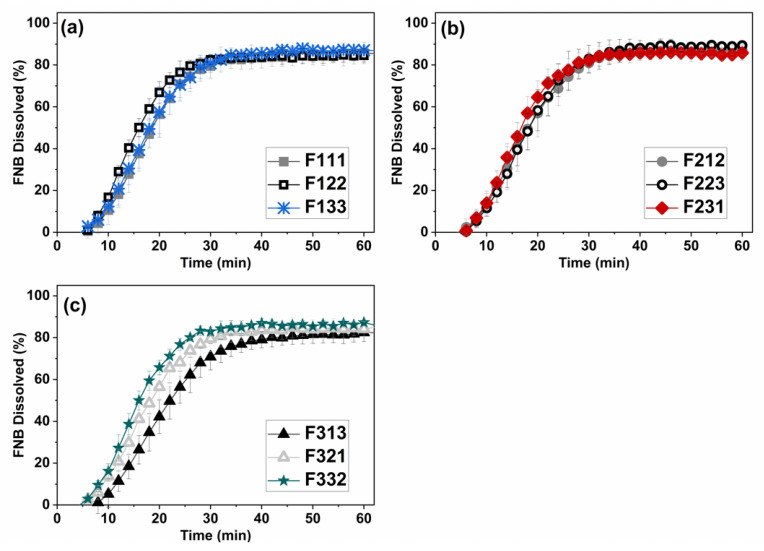
Dissolution profiles of dry films at ~23 wt% AR-FNB concentrations processed with: (**a**) Impeller mixer, (**b**) Planetary mixer, and (**c**) High-intensity vibrational mixer.

The dissolution profiles of the films containing 23 wt% FNB concentration are shown in Figure 7. In general, varying mixing conditions had no negative impact on drug release profile of the films. In all cases, immediate release behavior (>80% dissolved in ~30 min) was observed for all the films with the only exception being vibrational mixer at low mixing intensity and high mixing time that was expected to have more rigid-body motion than the mixing actions (F313) (see Figure 7c). The agglomeration issue discussed previously at poor mixing condition, F313, could be the source of a slower dissolution rate. This is in line with the previous reports on the impact of particle size on drug release behavior of poorly water-soluble drugs [2,6,8]. Overall, the important outcome was that regardless of the mixer type used, if mixing is adequate the film matrix was capable of enhancing the dissolution of poorly water-soluble FNB even with micron-sized drug particles without performing any surface modification. Having said that, it was also evident that as compared to using stabilized drug nano-suspensions [1,3] or dry-coated micro-sized particles [6], the use of as-received micro-sized drug powders led to incomplete release. Examination of the redispersion results presented in Figure 4 indicates that for all cases, the redispersed sizes were appreciably larger than the original primary particle sizes of the as-received drug, implying certain level of aggregation within dried-films. In contrast, when surface engineered drug particles were used after dry coating in a previous work, the redispersed size was nearly the same, see Table 2 in Zhang et al. [6]. The extent of agglomeration is likely to cause the extent of incomplete dissolution. Another reason for incomplete dissolution could be the intrinsic surface hydrophobicity of the drug particles. While that was addressed in previous work either via suspension stabilization [1,3] or dry coating [6], the present work did not employ such particle treatment. Overall, apart from a minor reduction in the extent of dissolution, these results demonstrate the role of proper mixing in manufacturing high quality films loaded with poorly water-soluble drugs. It is highly likely that dry coating using hydrophilic silica may further improve the product quality in conjunction with properly selected mixing conditions.

## 4. Conclusions

The type of mixer, mixing intensity including the mixing mechanism, and mixer CPPs were found to have a significant impact on slurry-cast film CQAs, in particular, on drug content uniformity. In general, mixing at higher intensities and longer processing times led to improved film CQAs even without using drug particles that were surface engineered, such as via dry coating with hydrophilic silica. Interestingly, high-shear planetary mixer, and even low-shear impeller mixer at higher rpm and processing time, outperformed high-intensity vibrational mixer. As a novelty, the drug homogeneity within film precursor was found to be an indicator of the subsequent drug CU in dry films, i.e., acceptable homogeneity was found to be a sufficient condition for acceptable dried-film drug CU. When the drug concentration was lower, i.e., 3.0 wt%, higher intensity and/or processing time were necessary for both the impeller and planetary mixers to achieve acceptable drug CU in dried films. At the lowest drug concentration, intended for low-dose products, i.e., ~70 µg/cm^2^, acceptable drug CU was achieved only via high-shear planetary mixer. Overall, very good film CQAs were achievable when the mixer type and their CPPs were properly selected even with as-received micro-sized powders of poorly water-soluble drug. However, use of the as-received powders did not promote full drug particle size recovery after film redispersion, indicating some extent of irreversible particle aggregation during film processing. That led to failing to achieve 100% drug dissolution even when the planetary mixer was used, demonstrating the need for surface engineering via dry coating [6]. Notwithstanding, judiciously selected mixer and its CPPs assured desirable film CQAs using low micro-sized drug powders without any particle pretreatment.

## Figures and Tables

**Table 1:** Composition of the precursor suspensions and target FNB concentrations in the dried film.

Run No.	wt% HPMC	wt% Glycerin	wt% Water	wt% FNB in Film Precursor	Target FNB Concentrations in Film (Wt%)
1	12	4	79.0	5.0%	22.73
2	12	4	83.5	0.50%	2.88
3	12	4	83.9	0.10%	0.59

**Table 2:** Mixing processing parameters via Taguchi L9 orthogonal array design of experiment.

Run No.	Mixer Type	Mixing Intensity	Mixing Time (Minute)
F111	Impeller	60 rpm	60
F122	Impeller	120 rpm	180
F133	Impeller	240 rpm	300
F231	Planetary	2000 rpm	1
F212	Planetary	1000 rpm	10
F223	Planetary	1500 rpm	15
F321	Vibrational	45 G	5
F332	Vibrational	60 G	10
F313	Vibrational	25 G	15

rpm: revolutions per minute, G: acceleration, m/s^2^.

**Table 3:** Average values and RSD% of dried-film thickness, drug concentration (wt%), drug content per unit area along with acceptance values (AV) for the films.

Run No	Thickness (μm)	RSD%	Wt% Fenofibrate	RSD%	Drug Content per Unit Area (mg/cm²)	RSD%	AV
F111	130.30	4.57	19.61	3.92	3.12	5.28	25.26
F122	132.63	3.59	22.59	2.10	3.75	4.31	5.02
F133	125.70	2.41	22.60	1.59	3.55	3.14	3.95
F231	133.85	4.60	21.98	1.10	3.61	5.05	4.72
F212	128.90	3.08	20.93	1.69	3.33	4.35	11.53
F223	130.05	1.82	21.58	2.48	3.45	2.02	10.26
F321	129.55	6.74	19.94	5.76	3.11	8.17	25.87
F332	131.50	4.20	22.00	1.47	3.58	4.87	6.07
F313	132.10	7.76	15.59	12.25	2.50	15.95	91.19

**Table 4:** Average values and RSD% of dried-films at varying drug concentrations (wt%), drug content per unit area along with acceptance values (AV).

Mixer Type	Theoretical wt% Drug	wt% Drug	RSD%	Drug Content (mg/cm²)	RSD%	AV
Impeller	22.73	22.60	1.59	3.55	3.14	3.95
	2.88	2.70	2.68	0.37	3.62	12.12
	0.59	0.49	10.11	0.07	10.89	45.48
Planetary	22.73	21.58	2.48	3.45	2.02	10.26
	2.88	2.75	1.17	0.36	2.66	6.28
	0.59	0.53	1.42	0.07	3.05	14.30

**Table 5:** Mechanical properties of 23 wt% FNB loaded films with fixed film thickness (0.12–0.13 mm).

Run No	Tensile Strength ± SD (MPa)	Elongation at Break ± SD (%)	Thickness ± SD (μm)
F111	16.1 ± 1.9	11.1 ± 2.4	124.7 ± 3.3
F122	17.0 ± 0.7	14.0 ± 3.0	129.0 ± 2.3
F133	17.4 ± 0.8	13.6 ± 0.7	126.5 ± 1.9
F231	15.1 ± 0.8	14.4 ± 2.1	124.5 ± 2.6
F212	16.1 ± 0.7	12.1 ± 1.0	125.8 ± 1.2
F223	17.7 ± 0.6	16.1 ± 1.2	123.4 ± 2.5
F321	15.5 ± 0.8	10.0 ± 1.4	125.0 ± 3.2
F332	18.6 ± 1.2	15.4 ± 1.8	127.7 ± 1.0
F313	17.8 ± 0.5	12.4 ± 2.9	124.0 ± 2.9

## Data Availability

Not applicable.

## Supplementary Materials

The following are available online at https://www.mdpi.com/article/10.3390/pharmaceutics13060812/s1, Table S1: Factors and levels of mixing processing conditions through Taguchi L9 orthogonal array, Table S2: RSD% values of drug amount (mg) per sample from precursor suspensions and dry films, Table S3: Response table of S/N ratios for RSD% of drug content uniformity based on drug dose (mg/cm^2^), Figure S1: Digital images of dry films containing ~23 wt% FNB concentration processed at different mixing conditions; F313, F321, and F223.
