# Supplementary material for: Impact of Mixing on Content Uniformity of Thin Polymer Films Containing Drug Micro-Doses
Source: Pharmaceutics. 2021 May 29;13(6):812. doi: 10.3390/pharmaceutics13060812 (PMC8229899; doi:10.3390/pharmaceutics13060812)
Supplement: Supplementary file 1 [file pharmaceutics-13-00812-s001.zip › pharmaceutics-1236423-supplementary.pdf]

pharmaceutics

Supplementary Materials: Impact of Mixing on Content Uniformity of Thin Polymer Films Containing Drug Micro-Doses

Guluzar G. Buyukgoz, Jeremiah N. Castro, Andrew E. Atalla, John G. Pentangelo, Siddharth Tripathi and Rajesh N. Davé

Table S1. Factors and levels of mixing processing conditions through Taguchi L9 orthogonal array.

Factors	Level 1	Level 2	Level 3
Mixer Type	Impeller	Planetary	Vibrational
Mixing Speed	Low	Medium	High
Mixing Time	Slow	Intermediate	Fast

Table S2. RSD% values of drug amount (mg) per sample from precursor suspensions and dried-films.

Run	RSD% (Precursor)	RSD% (Dried-film)
F111	19.01	5.28
F122	6.55	4.31
F133	1.59	3.14
F231	2.71	5.05
F212	9.69	4.35
F223	4.04	2.02
F321	17.69	8.17
F332	6.89	4.87
F313	27.83	15.95

Table S3. Response table of S/N ratios for RSD% of drug content uniformity based on drug dose (mg/cm^2).

Level	Type	Intensity	Time
1	−12.36	−17.09	−15.59
2	−10.98	−12.35	−13.07
3	−18.68	−12.58	−13.37
Delta	7.7	4.75	2.52
Rank	1	2	3

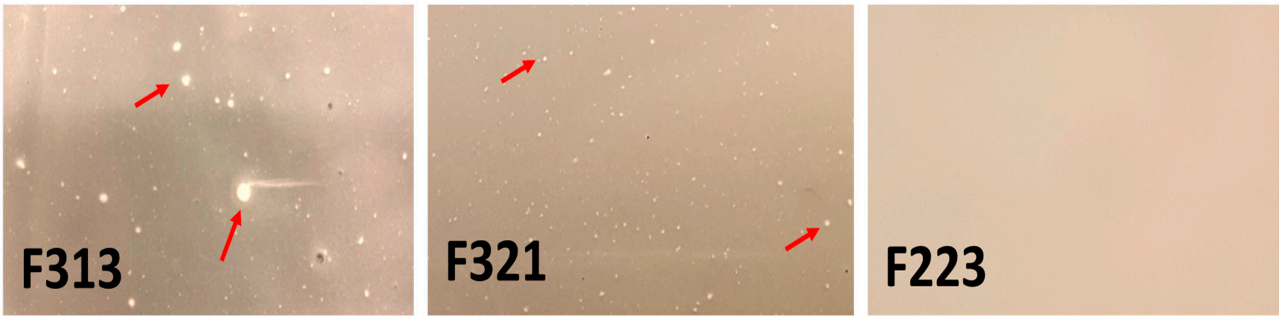

Figure S1. Digital images of dried-films containing ~23 wt% FNB concentration processed at different mixing conditions; F313, F321, and F223.
